# Pinewood Biochar as an Affordable Adsorbent for Short- and Medium-Chain PFAS Removal from Contaminated Water

**DOI:** 10.3390/molecules31071100

**Published:** 2026-03-27

**Authors:** Giulia Simonetti, Patrizia Di Filippo, Donatella Pomata, Carmela Riccardi, Francesca Buiarelli, Stefano Parisi, Marco Petrangeli Papini, Marta Senofonte, Laura Lorini

**Affiliations:** 1 Department of Chemistry, University of Rome “La Sapienza”, 00185 Rome, Italy; francesca.buiarelli@uniroma1.it (F.B.); parisi.stefano99@gmail.com (S.P.); marco.petrangelipapini@uniroma1.it (M.P.P.); laura.lorini@uniroma1.it (L.L.); 2 Department of Technological Innovations and Safety of Plants, Products and Anthropic Settlements, National Institute for Insurance against Accidents at Work (INAIL), 00144 Rome, Italy; p.difilippo@inail.it (P.D.F.); d.pomata@inail.it (D.P.); ca.riccardi@inail.it (C.R.)

**Keywords:** biochar, pinewood-derived biochar, PFAS removal, short-chain PFAS, continuous-flow adsorption, sustainable adsorbents

## Abstract

The present paper investigates the adsorption performance of pinewood-derived biochars produced at two pyrolysis temperatures (850 °C, PW-A; 1000 °C, PW-B), including sieved fractions (PW-A1 and PW-A2) and a functionalized variant (PW-C), for the removal of five short- and intermediate-chain PFASs (PFBA, PFBS, PFHxA, PFHxS, and GenX) from water under continuous-flow conditions. Adsorption behavior was evaluated using Freundlich and Hill isotherm models. The Hill model provided a superior fit for most PFAS–adsorbent systems, highlighting the importance of cooperativity effects, particularly for short-chain PFASs. In single-compound experiments, PFBS and GenX showed the highest adsorption capacities (up to 82.3 and 68.5 mg g^−1^), while PFBA and PFHxA exhibited the lowest. Among the tested materials, biochar produced at 1000 °C (PW-B) consistently demonstrated the highest adsorption efficiency. Compared to activated carbon, PW-B showed comparable performance for PFBA, PFBS, PFHxA and PFHxS and significantly better performance for GenX. In mixed-PFAS systems, competitive effects reduced adsorption capacity and cooperativity. Sulfonic PFASs showed higher affinity than carboxylic PFASs, following the trend PFHxS > PFBS > PFHxA > PFBA. Overall, the results demonstrate that waste-derived biochar represents a low-cost and sustainable alternative for PFAS removal in realistic water-treatment scenarios, supporting scalable solutions aligned with global environmental goals.

## 1. Introduction

Per- and polyfluoroalkyl substances (PFASs) are a large class of synthetic, highly fluorinated compounds characterized by their persistent nature and remarkable chemical stability, attributed to the strong carbon–fluorine (C-F) bonds. These compounds, first synthesized in the 1950s, have found extensive applications across various industries due to their unique properties, including waterproofness and resistance to grease and thermal and biological degradation. However, their widespread use has led to significant environmental contamination, with PFASs now being detected in water, soil, and sediments. Since these pollutants pose serious risks to human health and ecosystems, being linked to endocrine disruption, carcinogenesis, and metabolic dysfunction, regulations and guidelines have been implemented globally to limit their concentrations in the environment and drinking water. In this regard, many long-chain PFASs are banned or restricted in several countries due to their health impact and have been replaced by short and ultra-short PFASs [1]. However, the environmental occurrence of short-chain PFASs is also of concern since they are as persistent as long-chain PFASs. In fact, due to their higher mobility and low adsorption potential in water bodies compared to long-chain compounds, short-chain PFASs could significantly affect drinking water supplies, increasing human exposure to such compounds. Increasing concentrations of short-chain PFASs, such as PFBA, PFBS, and PFHxA, have already been detected all over the world, both in water and sediments. Furthermore, because of their physicochemical properties, they are also more prone to long-range transport and monitoring data confirm their presence in remote environments and across biotic and abiotic systems. Therefore, after the phase-out of long-chain PFASs, the possible outcome of the increasing production of short-chain PFASs sounds quite alarming, both for the ecosystem and human health [2,3]. In addition, remediation technologies currently suitable for long-chain PFASs have often proven to be ineffective for short-chain PFASs. In this regard, among the many reclamation techniques available [4], adsorption techniques are emerging as a key strategy in water treatment, even though short-chain PFASs are not easily removed by adsorption, since they do not readily bind to particles and remain soluble in the water phase, highlighting the need for innovative methodologies [5]. In this regard, the study of PFAS adsorption using novel adsorbent materials, carried out with continuous-flow systems such as a continuously stirred tank reactor (CSTR), has proven to be a valid alternative approach for long-chain PFASs [6,7]. The continuous-flow system is a promising alternative as it can be considered a hybrid batch and column experiment and therefore it can provide a more dynamic and representative assessment of adsorption processes. Furthermore, this kind of system can provide consistent and controlled experimental conditions, along with reduced volumes and time required for laboratory tests. In the present study, a continuous-flow reactor system was employed to evaluate the adsorption of short- and intermediate-chain PFASs (e.g., PFBA, PFBS, GenX, PFHxA, and PFHxS), investigating both novel and conventional adsorbents used in remediation (pinewood biochar at a pyrolysis temperature of 850 °C and 1000 °C; activated carbon). Among the different biochar feedstocks, pinewood represents an attractive precursor due to its wide availability, renewable nature, and its ability to generate highly carbonized structures with well-developed porosity when pyrolyzed at high temperatures. These characteristics make pinewood-derived biochar a promising low-cost adsorbent for hydrophobic organic contaminants such as PFASs.

This configuration not only enabled direct comparison with widely used materials, such as activated carbon but also offered critical insights into the adsorption performance of sustainable alternatives like functionalized pinewood biochar. Furthermore, the present paper highlights the role of pinewood biochar that, derived from renewable biomass through the pyrolysis process, offers a low-cost, low-impact option for PFAS remediation. Therefore, by integrating innovative testing methodologies with waste-derived materials, this work contributes to the development of scalable, sustainable water treatment technologies aligned with resource recovery strategies and circular economy approaches for environmental protection.

## 2. Results and Discussion

### 2.1. Kinetic Tests

**Figure 1:**
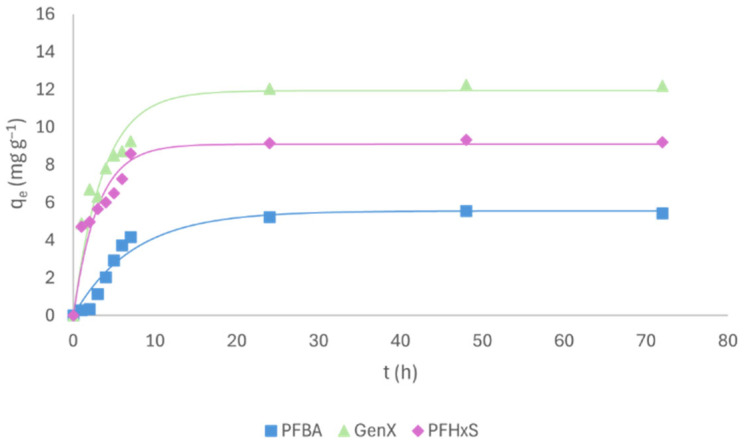
Kinetic tests of PFBA, GenX and PFHxS.

Kinetic tests were carried out to determine the contact time required to reach equilibrium. As an example, Figure 1 shows the kinetic curves for PFBA, GenX and PFHxS on PW-B and Table S1 reports the related parameters. As shown by correlation coefficients, the first-order kinetic model fitted the data well. Similar kinetic behavior was also observed for the other compounds and the other adsorbents as already reported in [6,8]. It can be noted that the equilibrium was reached after 24 h; furthermore, the PFBA amount adsorbed in this time was lower than that of GenX and PFHxS, highlighting a faster removal capacity of PW-B for the two intermediate-chain PFASs as indicated by the higher k values (Table S1). Moreover, as reported in the literature, the intra-particle diffusion effect could explain the low adsorption rate observed in the initial stage of the curve, acting as a rate-controlling step [9].

### 2.2. Equilibrium Tests

The adsorption capacity of PW materials for the target PFAS was estimated with Freundlich and Hill models. In addition to the Freundlich model, the Hill model was applied to describe the adsorption behavior. This model is particularly suitable for evaluating potential cooperative adsorption effects, where the adsorption of one molecule may facilitate the adsorption of additional molecules on neighboring sites. Such behaviour may occur with PFASs due to their amphiphilic nature and the possibility of intermolecular interactions at the adsorbent surface. The Freundlich model has been demonstrated to effectively characterize the adsorption behavior of long-chain PFASs in equilibrium studies [10]. Conversely, in the present study, short- and medium-chain PFAS adsorption behavior is more accurately represented by the Hill model, where a cooperative factor is considered through the n_H_ parameter. The cooperative process plays a crucial role, and the binding of one molecule seems to promote the binding of others through aggregation or interactions between the adsorbed molecules. Table 1 shows the parameter and correlation coefficient values of the best-fitting model to describe isotherms. These parameters and the PFAS compound amount adsorbed at a given specific equilibrium concentration served as a basis to compare the performance of the biochars under investigation.

Therefore, the interpretative statements were developed by comparing the behavior of the different biochars and considering the functional groups of the investigated compounds. Based on these observations, qualitative hypotheses were formulated to explain the differences in PFAS adsorption among the various biochars studied.

#### 2.2.1. PFBA and PFBS

**Figure 2:**
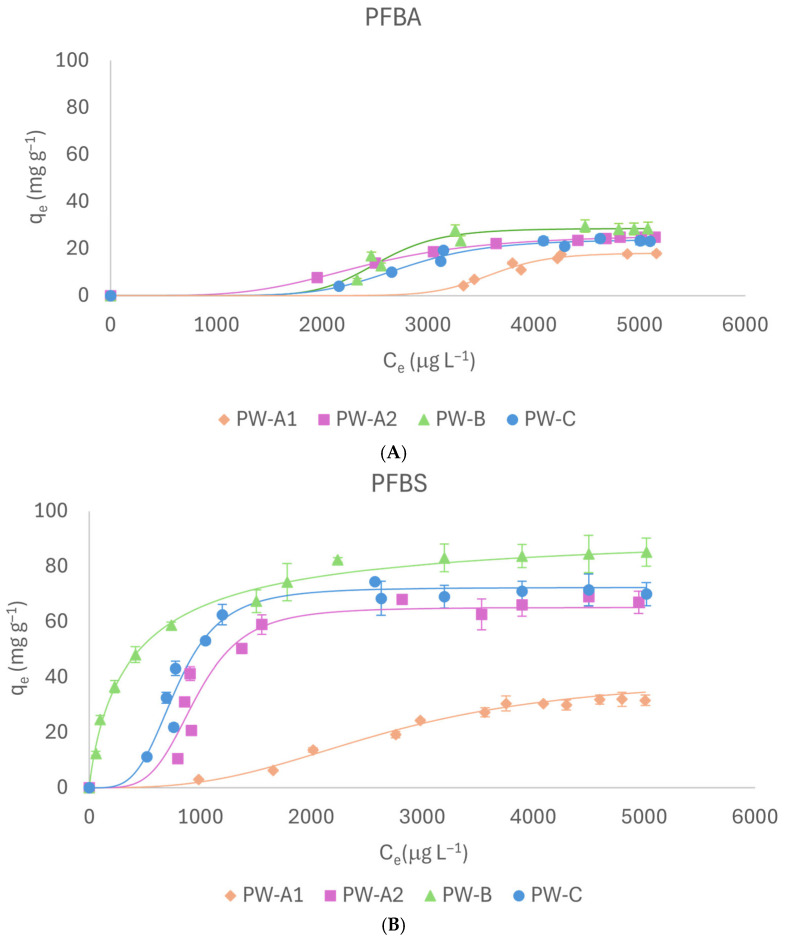
Adsorption isotherms of PFBA (**A**) and PFBS (**B**).

Figure 2 shows adsorption isotherms of PW materials. For PFBA, both the sieved and unsieved PW-A exhibited similar adsorption capacities (17.95 mg g^−1^ for PW-A1; 22.09 mg g^−1^ for PW-A2). In the case of PFBS, PW-A1 achieved an adsorption capacity (30.47 mg g^−1^) slightly exceeding that observed for PFBA, whereas PW-A2 demonstrated a significantly enhanced adsorption capacity (62.63 mg g^−1^). The greater surface area of PW-A2 and the PFBS sulfonic group probably improved the adsorption process, as also shown by recent studies [11]. PW-B proved to have a higher adsorption capacity for both PFBA (27.59 mg g^−1^) and PFBS (82.33 mg g^−1^), with a stronger affinity for PFBS than for PFBA. This enhanced performance can again be attributed to the sulfonic group, increased surface area, and greater pore volume within PW-B, which overall facilitate more effective interactions between the adsorbate and adsorbent. Regarding PW-C, the adsorption capacities measured for PFBA and PFBS were 23.24 mg g^−1^ and 71.5 mg g^−1^, respectively, thereby highlighting the non-negligible function of the sulfonic group. Indeed, the process appears to be strongly influenced by electrostatic interactions between PW-C’s positively charged surface and PFASs’ negatively charged hydrophilic groups [12]. This is particularly significant for short-chain PFASs, where the hydrophilic character increases as the length of the hydrophobic perfluorinated carbon chain decreases [13]. Furthermore, greater resistance to adsorption of PFBA across all tested biochars may be attributed to the reduced steric hindrance and lower electronegativity of the carboxyl group. These characteristics enable PFBA molecules to more readily diffuse out from the pores present on the biochar’s active surface. Additionally, PFBA’s physicochemical properties, such as high solubility and reduced hydrophobicity, further constrain its adsorption efficiency. This is further corroborated by the n_H_ values for PFBA, which showed the highest positive values (ranging from 4.11 to 13.18), suggesting that adsorption at one site may enhance subsequent adsorption at neighboring sites via hydrophobic interactions, π-π stacking, and PFAS cluster formation, thereby promoting multilayer adsorption. Nonetheless, this cooperative effect appears to become significant only after surpassing a molecular interaction threshold. In addition, the higher K_d_ values for PFBA reinforce its reduced affinity for the adsorbents examined, as higher K_d_ values are indicative of weaker binding strength.

#### 2.2.2. PFHxA and PFHxS

**Figure 3:**
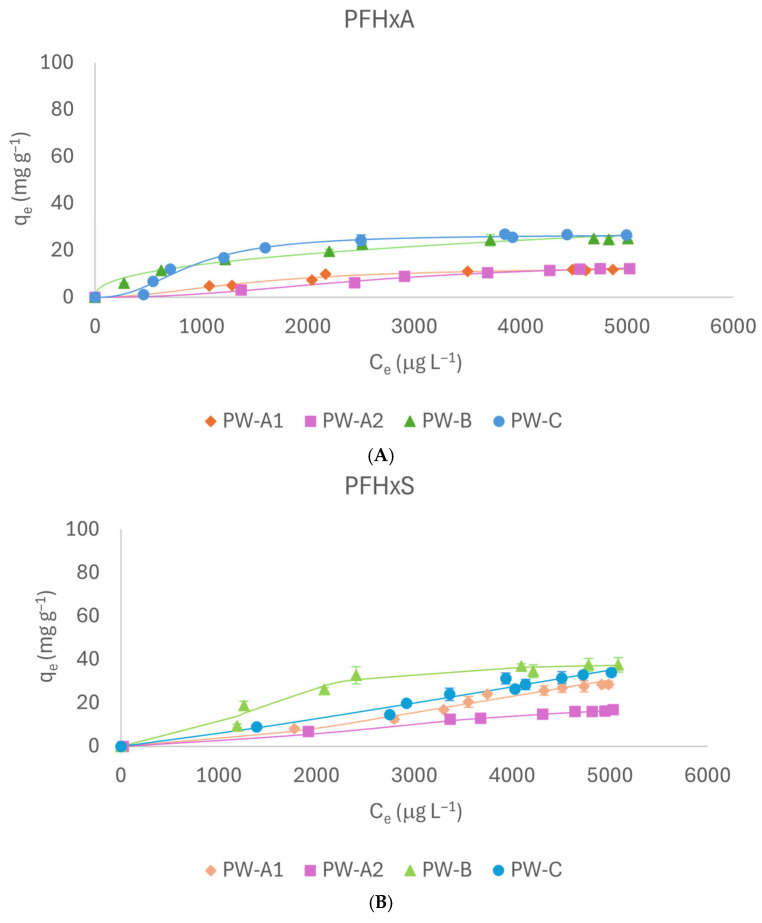
Adsorption isotherms of PFHxA (**A**) and PFHxS (**B**).

Figure 3A,B show adsorption isotherms of PFHxA and PFHxS, respectively. The Hill model fitted the data well (R^2^ ≥ 0.98) with the exception of the PW-C adsorbent, for which the Freundlich model performed better. The lowest adsorption capacities were observed for PFHxA on PW-A. Specifically, PW-A1 and PW-A2 exhibited the most limited adsorption, with values of 11.89 mg g^−1^ and 12.17 mg g^−1^, respectively. PW-B and PW-C showed higher adsorption capacities (24.93 mg g^−1^ and 26.54 mg g^−1^, respectively). This enhanced performance is likely attributable to their greater surface area and increased pore volume. The superior affinity of PW-B and PW-C is further highlighted by lower K_d_ and higher Q_max_ values (Table 1), which also indicate an increased binding capacity. Additionally, Hill exponent values exceeding 1 show positive cooperativity, implying that the adsorption of one molecule promotes the subsequent binding of others.

PFHxS displayed higher q_e_ values compared to its carboxylic counterpart, as already observed for PFBA and PFBS. In detail, the highest values were detected for PW-B (36.9 mg g^−1^) and PW-C (31.2 mg g^−1^), highlighting similar performances. Accordingly, for these compounds, the nature of the sulfonic groups also positively influences the adsorption process since stronger electrostatic interactions can be established, increasing the affinity between the material and molecules [14,15]. However, the lower affinity of these PFASs for the study adsorbents compared to the short-chain ones is likely be due to the longer chain length that, for increased steric hindrance, causes a smaller number of favorable interactions between the molecules and the adsorbent. Consequently, interactions with other molecules are weaker, limiting the cooperativity in hydrophobic or electrostatic interactions.

#### 2.2.3. GenX

**Figure 4:**
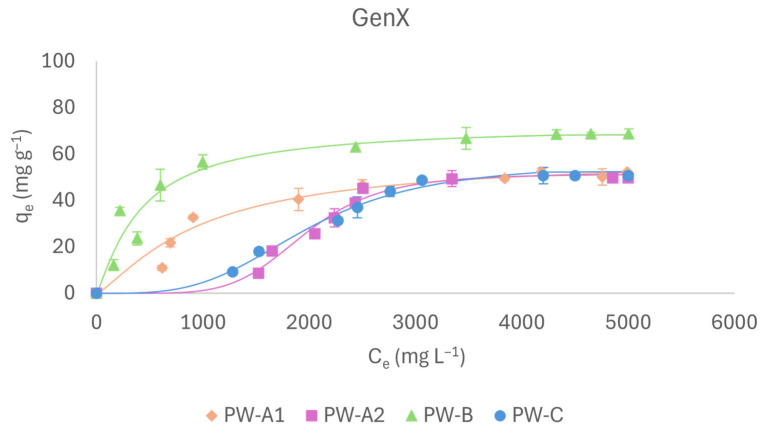
Adsorption isotherms of GenX.

GenX adsorption is peculiar due to its amphiphilic nature and distinctive chemical branched structure. The experimental tests proved that the Hill model fits all adsorbents well (R^2^ > 0.98, Table 1), and q_e_; values were consistently high for all biochars (Figure 4). Specifically, both size fractions of PWA exhibited comparable adsorption capacities (about 55 mg g^−1^) that were mostly higher than those shown by the other PFASs. Regarding PW-B, it displayed the highest adsorption capacity (68 mg g^−1^), likely due to a combination of hydrophobic interactions and a larger specific surface area. The more branched structure of GenX decreases its polarity and enhances its affinity for apolar surfaces such as that of PW-B, consistent with the lowest K_d_ value observed among the materials (Table 1). In addition, PW-B’s nH value, which was lower than the other biochars, may be associated with its structural homogeneity and extensive apolar surface area, where hydrophobic interactions dominate over cooperative molecule–molecule effects. Finally, the adsorption capacity of PW-C does not differ substantially from that of the non-functionalized PW-A. Like other short-chain PFASs discussed previously, the initial adsorption of GenX can be attributed to electrostatic interactions between its negatively charged hydrophilic group and the positively charged biochar surface. Moreover, micellar interactions may significantly influence the process, as the amphiphilic nature of GenX may promote micelle formation once its concentration exceeds the critical micelle concentration (CMC) [16,17]. However, the adsorption of micellar aggregates—characterized by larger steric hindrance—can induce pore blockage and reduce overall adsorption efficiency.

### 2.3. Biochars vs. Activated Carbon

**Figure 5:**
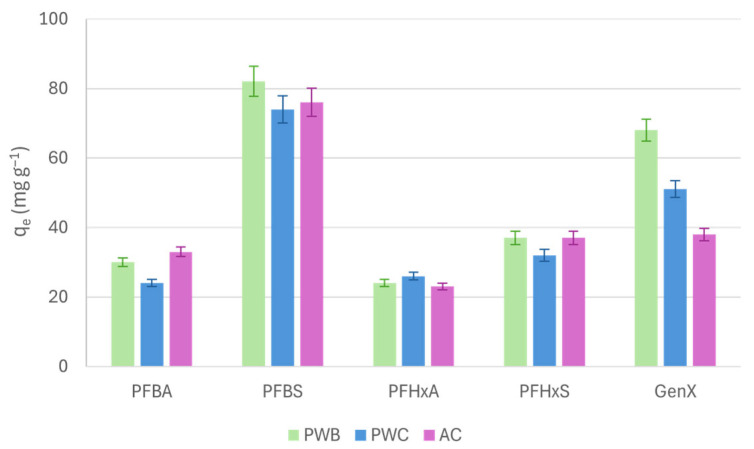
Comparison of PFAS q_e_ values for PW-B, PW-C and AC.

As already observed in previous work [7], in the case of short-to-intermediate PFASs, PW-B and PW-C were also the most promising adsorbents in terms of removal efficiency. For this reason, their adsorption capacities were compared to AC, to evaluate their performances in comparison with the most common material used in the PFAS remediation field [18]. Further details on AC adsorption behavior and the models applied can be found in the Supplementary Materials (Figure S1 and Table S2). Figure 5 shows the maximum q_e_ values observed for PW-B, PW-C, and AC for each PFAS. In general, PW-B and PW-C showed comparable performances to AC for PFBA, PFBS, PFHxA and PFHxS. This could be ascribed to the high surface area combined with the predominantly non-polar domain in the case of PW-B and the greater number of positively charged sites available for interaction in PW-C. However, in the case of GenX, the adsorption efficiency was extremely improved. The reduced affinity of GenX for AC could probably be explained by the presence of a higher number of micropores compared to PW-B. This may have led to a pore-blocking effect due to its large chemical structure, resulting in monolayer adsorption preventing further molecule aggregation [18].

Therefore, a greater abundance of mesoporous structures of PW-B can enhance the diffusion and adsorption of GenX molecules. Conversely, the predominantly microporous architecture of activated carbon may restrict the accessibility of adsorption sites for GenX. In addition, the presence of surface functional groups on functionalized biochar (PW-C) may further facilitate specific interactions with GenX molecules. As already stated before, the adsorption resistance of PFHxA and PFHxS, also evidenced in the case of AC, could be explained by a combination of steric hindrance from the chain length and reduced cooperativity between molecules. However, the adsorption capacities of these carbonaceous materials show similarities and in the case of GenX, the PW-B exceeded the AC performance.

Table 2 compares the data from this work with those obtained by other authors.

The adsorption capacities of the pinewood-derived biochars varied depending on the PFAS compound and were, in most cases, comparable to or higher than those reported in the literature for activated carbon. Higher q_e_ values were observed for PFBA and PFBS in PW tests, while PFHxA showed values comparable to those reported in previous studies. For other compounds, i.e., PFHxS and GenX, the adsorption capacities fell within or slightly below the literature ranges. Therefore, these results suggest that pinewood-derived biochar produced under more severe pyrolysis conditions can achieve adsorption performances similar of those of conventional activated carbon, particularly for short-chain PFASs such as PFBS.

### 2.4. PFAS Mixture

**Figure 6:**
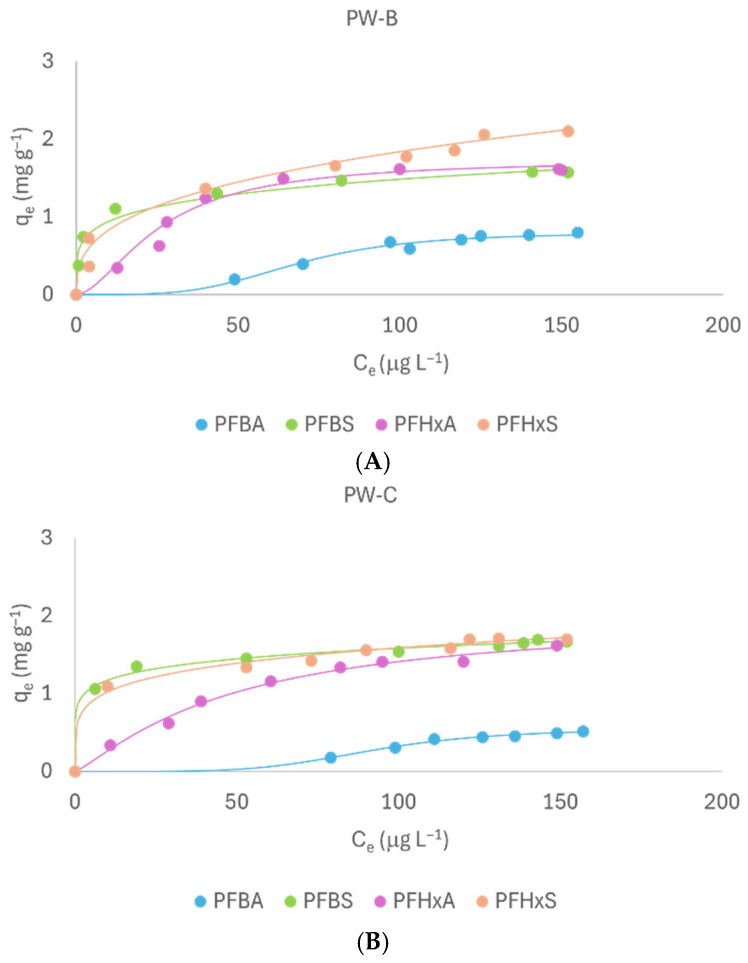
Adsorption isotherms of mixture of PFBA, PFBS, PFHxA and PFHxS on PW-B (**A**) and PW-C (**B**).

Figure 6 shows the adsorption isotherms for a mixture of short- and intermediate-chain PFAS on PW-B (Figure 6A) and PW-C (Figure 6B). The adsorbents PW-B and PW-C were selected for their superior adsorption capacities observed in preliminary tests using individual PFAS compounds. The PFAS mixture consisted of PFBA, PFBS, PFHxS, and PFHxA with each analyte present at an initial concentration of 0.150 mg L^−1^. PFAS concentrations were selected to enable reliable determination of adsorption parameters and to investigate adsorption performance under controlled laboratory conditions. Although this concentration was considerably lower than that utilized in adsorption tests with individual compounds, this level was selected to more accurately reflect environmental pollution scenarios and to maintain compatibility with analytical instrumentation limitations. GenX was omitted from this mixture because its fluoroether structure and physicochemical properties distinguish it from the homologous series of sulfonic and carboxylic acid PFASs. Since the objective of the mixed-PFAS experiments was to elucidate the influence of chain length and head-group chemistry within structurally comparable compounds, including GenX would not have contributed to this comparative assessment and could have confounded data interpretation. The adsorption capacity of the investigated materials toward the selected PFAS was evaluated by applying either the Freundlich or Hill models. Table 3 shows the parameters of the best-fitting model for each material. All models exhibited high goodness-of-fit (R^2^ > 0.98). No significant differences in maximum adsorption capacities were observed between PW-B and PW-C. Both materials exhibited higher q_e_ values for sulfonic PFASs (PFBS: 1.56 mg g^−1^ for PW-B and 1.69 mg g^−1^ for PW-C; PFHxS: 2.15 mg g^−1^ for PW-B and 1.80 mg g^−1^ for PW-C) compared to carboxylic PFASs (PFBA: 0.77 mg g^−1^ for PW-B and 0.52 mg g^−1^ for PW-C; PFHxA: 1.60 mg g^−1^ for both), confirming the stronger affinity of sulfonic groups.

This trend underscores the dominant role of electrostatic interactions, which outweigh hydrophobic contributions from chain length. PFBA showed the lowest adsorption, likely due to its short chain and less electronegative carboxyl group. Model fitting revealed distinct adsorption behaviors within the mixture. PFBS and PFHxS were best described by the Freundlich model, whereas PFBA and PFHxA followed the Hill model for both adsorbents. The low n values obtained for sulfonic PFASs in the Freundlich model suggest heterogeneous adsorption sites and possible multilayer formation, consistent with their strong electrostatic interactions. This divergence from single-compound trends may reflect competitive effects in mixed solutions, which reduce the cooperative adsorption observed in isolated tests. PFBA exhibited the highest n_H_ values, confirming its strong cooperative behavior, while PFHxA showed a weaker sigmoidal trend for PW-C, consistent with its reliance on hydrophobic rather than electrostatic interactions. Overall, short-chain PFAS adsorption appears to be hindered by intermediate-chain compounds, likely due to competitive displacement by PFHxA and PFHxS [23]. In summary, the overall adsorption affinity within the mixture followed the order PFHxS > PFBS > PFHxA > PFBA. Consistent with single-compound studies, the sulfonic group exerted a stronger influence on adsorption than chain length. These findings highlight the importance of considering competitive effects in real-world contamination scenarios, where multiple PFASs coexist and may reduce overall adsorption efficiency.

## 3. Materials and Methods

### 3.1. Chemicals and Materials

Five per- and polyfluoroalkyl substance (PFAS) standards, each with short to medium chain lengths (ranging from C4 to C6) and distinct functional groups, were selected for investigation: perfluorbutanoic acid (PFBA), perfluobutanesulfonic acid (PFBS), 2,3,3,3-tetrafluoro-2-(1,1,2,2,3,3,3-heptafluoropropoxy)propanoic acid (GenX), perfluorohexanoic acid (PFHxA), and perfluorohexanesulfonic acid (PFHxS) (LGC standard Ltd., Milano, Italy). Table S3 shows the main proprieties of the target PFASs.

For chemical analysis, two mass-labeled internal standards (ISs) (i.e., 13C4-PFOS and 13C4-PFBA) were employed. Biochars were supplied by Burkhardt GmbH (Mühlhausen Germany). Specifically, these adsorbents were produced through pinewood (PW) gasification, at two temperature conditions: 850 °C (PW-A) and 1000 °C (PW-B). The biochar generated at 850 °C was further sieved to obtain two separate fractions: particles greater than 200 μm (PW-A1) and those less than 200 μm (PW-A2). The largest fraction, PW-A2, underwent additional functionalization (PW-C). The activated carbon (AC) was FILTERCARB PHA, purchased from CARBONITALIA S.r.l (Vezzano Ligure, Italy). Comprehensive characterization details of the biochars are available in a previous paper [7]. A summary table (Table S4) that reports biochar characterization data of a previous paper [7] is included in the Supplementary Material.

### 3.2. Flow-Through System Setup

System setup scheme and details can be found in a previous study [6]. Briefly, adsorption tests were carried out using columns packed with the selected adsorbents (PW-A1, PW-A2, PW-C, PW-B, and AC) and inert glass beads. Each column contained approximately 20.0 ± 0.5 mg of adsorbent. To prevent solid particle movement within the system, 0.20 µm non-woven fabric filters were placed at both ends of the columns. During assembly, the columns were vortexed with a mixer (Velp Scientifica, Usmate Velate, Italy) to promote a uniform distribution of the materials. A peristaltic pump (Baoding Shenchen Precision Pump Co., Ltd., Formello, Italy) maintained a steady flow rate of 0.6 mL min^−1^. The pump circulated the PFAS-contaminated solution in a closed-loop system, moving it from the storage tank, through the column, and back. The storage tank was equipped with a magnetic stirrer (VELP Scientifica, Usmate Velate, Italy), which provided continuous mixing that prevented concentration gradients. Before each adsorption test, the system was operated in up-flow mode with deionized water for 24 h at the target flow rate to verify system integrity and to check for any leaks.

### 3.3. Functionalization

PW-A1 underwent a functionalization treatment using the cationic surfactant Cetyltrimethylammonium bromide (CTAB) as well. CTAB was selected as the cationic surfactant due to its ability to introduce positively charged surface groups on carbonaceous materials, which can enhance electrostatic interactions with anionic PFAS species. Further details regarding the complete methodology are available in a previous publication [7]. Briefly, the process consisted of dispersing 1.5 g of biochar in 150 mL of deionized water containing 40 mg of CTAB. This mixture was subjected to continuous stirring for 24 h. Afterwards, the suspension was filtered, and the retained solids were dried at 120 °C for 24 h.

### 3.4. HPLC/MS-MS

The analytical protocol for PFAS determination in water samples was carried out as described in Petrangeli et al. [8]. Briefly, each sample was analyzed using an Agilent 1290 high-performance liquid chromatography (HPLC) system coupled with an Agilent G6460 triple quadrupole mass spectrometer (QqQ MS/MS) (Agilent Technologies, Toronto, CA, USA) equipped with an electrospray ionization source (ESI) operating in negative mode. Analyte separation was performed on a Waters Xbridge BEH (Ethylene Bridged Hybrid) C_18_ column (2.5 μm × 2.1 mm × 100 mm) (Waters, Milford, MA, USA), in combination with an Ultra C_18_ delay column (5 µm × 30 mm × 2.1 mm) (Restek, Bellefonte, PA, USA). The injection volume was set at 5 μL, with a controlled flow rate of 0.2 mL min^−1^. The mobile phase consisted of 15 mM ammonium acetate in ultra-pure deionized water (MilliQ^®^) (A) and methanol (B).

### 3.5. Kinetic Tests

Kinetic experiments were performed in accordance with the methodology outlined in a previous publication [8], at room temperatures and at a near-neutral pH, utilizing the pseudo-first-order kinetic model. The PFAS amount adsorbed at equilibrium at a given time (q_t_) was calculated using the following equation:

(1) $$q_{t}=\frac{\left(C_{0}-C_{t}\right)V}{m}$$

where C_0_ (µg L^−1^) represents the initial concentration of the individual PFAS compound, while C_t_ (µg L^−1^) is its concentration at sampling time t (h), V (L) is the total solution volume, and m (mg) is the mass of the adsorbent employed.

### 3.6. Modeling Calculation

Equilibrium adsorption experiments were carried out to assess the adsorption capacities of the investigated materials and to evaluate their selectivity towards PFAS compounds. All experiments were performed in triplicate. The relative standard deviation (RSD) ranged from 2% to 12%; these values are considered acceptable given the inherent variability of the experimental system. As described in Senofonte, 2025 [6], to interpret the equilibrium data, two isotherm models, the Hill and Freundlich equations, were employed. Both models were systematically applied to each dataset to determine which provided the most accurate representation of the experimental results. The Hill isotherm, described in Equation (2), is expressed as follows:

(2) $$q_{e}=Q_{max}\;\frac{C_{e}^{n_{H}}}{K_{D}+C_{e}^{n_{H}}}$$

where C_e_ is the equilibrium concentration of the adsorbate (mg L^−1^), q_e_ is the amount of PFAS adsorbed per unit mass of sorbent (mg g^−1^), and Q_max_ represents the maximum adsorption capacity at saturation (mg g^−1^). K_D_ is the Hill constant, defined as $K_{d}^{n_{H}}$, where K_d_ is the dissociation constant (mg L^−1^) and corresponds to residual PFAS concentration at the half-saturation of sites (i.e., K_d_ = $C_{eq}^{50}$). n_H_ is the Hill cooperativity coefficient, indicating the nature of interactions between adsorbent sites and PFASs: n_H_ > 1 reflects positive cooperativity in binding, n_H_ = 1 denotes non-cooperative or hyperbolic binding, and n_H_ < 1 suggests negative cooperativity. The Hill equation, in its generalized form incorporating $K_{d}^{n_{H}}$, is a robust tool for characterizing cooperative adsorption phenomena and is widely applicable across biochemical and environmental systems, including the adsorption of organic pollutants [24,25]. In this form, the model has been adapted to describe adsorption phenomena, like the Langmuir isotherm but incorporating cooperativity effects with enhanced versatility for complex binding scenarios [26]. The empirical Freundlich isotherm, which assumes adsorption onto heterogeneous surfaces, is given in Equation (3):

(3) $$q_{e}=K_{F}C_{e}^{1/n}$$

where K_F_ is the Freundlich constant and n is a dimensionless parameter greater than zero, reflecting adsorption efficiency as concentration increases.

## 4. Conclusions

This study evaluated the adsorption performance of pinewood-derived biochars produced at two pyrolysis temperatures (850 °C, PW-A; 1000 °C, PW-B), including sieved and unsieved fractions (PW-A1 and PW-A2) and a functionalized variant (PW-C), for the removal of five short- and intermediate-chain PFASs (PFBA, PFBS, PFHxA, PFHxS, and GenX) under continuous-flow conditions. The adsorption model study indicated that the Hill equation provided the best fit for most systems, revealing positive cooperativity (nH > 1) across all PFAS–adsorbent combinations and providing useful suggestions for their full-scale application. Single-compound tests showed that PFBS and GenX exhibited the highest adsorption capacities, while PFBA and PFHxA were the least adsorbed, reflecting the combined influence of sulfonic group electronegativity and hydrophobic interactions. PW-B consistently achieved superior removal efficiency, even outperforming activated carbon for GenX, whereas PW-A showed lower performance, likely due to reduced surface area and pore volume. Functionalization (PW-C) further enhanced adsorption, indicating contributions from electrostatic and micellar interactions in addition to hydrophobic forces. The stated hypotheses are intended as a qualitative interpretation of experimental results that require further tests to be confirmed. The PFAS mixture experiments revealed competitive effects that reduced overall adsorption and attenuated cooperativity compared to single-compound tests. Sulfonic PFASs maintained higher affinity than carboxylic PFASs, with the observed trend PFHxS > PFBS > PFHxA > PFBA, confirming the dominant role of electrostatic interactions over chain length. Therefore, in full-scale studies, the role of different compounds and their affinity for absorbents should be considered. In addition, the continuous-flow configuration proved to be a more realistic and scalable approach than traditional batch tests, offering dynamic conditions that better simulate operational environments. Future research should address regeneration and reuse strategies for biochar, evaluate cost-effectiveness at an industrial scale, and explore integration with complementary technologies such as advanced oxidation or membrane filtration. Overall, this work demonstrates the feasibility of using waste-derived biochar as a low-cost, low-impact alternative to conventional adsorbents for PFAS remediation, providing actionable insights for optimizing treatment under competitive conditions and contributing to sustainable water treatment solutions aligned with emerging regulatory targets. Furthermore, the results can provide actionable insights for optimizing adsorbent design and improving PFAS removal efficiency under competitive conditions. Therefore, future research should focus on strategies to optimize adsorption under these conditions, particularly in systems containing both short- and long-chain PFASs, which is critical for designing effective water treatment technologies and understanding competitive interaction mechanisms.

## Figures and Tables

**Table 1:** Adsorption parameters of Hill (Q_max_, K_D_, n_H_, and R^2^) and Freundlich (K_F_, *n*, and R^2^) models.

PFAS	Biochar (PW)	Parameters of Hill Model			
		Q_max_	K_D_	n_H_	R^2^
PFBA	A1	18.14	3603	13.18	0.984
	A2	26.01	2421	4.111	0.999
	B	28.60	2538	8.765	0.976
	C	23.95	2766	6.848	0.988
PFBS	A1	40.21	2692	2.931	0.995
	A2	65.14	961.3	4.391	0.986
	B	94.74	399.4	0.864	0.983
	C	72.38	786.4	3.874	0.976
PFHxA	A1	13.04	1453	3.065	0.992
	A2	15.48	2696	2.251	0.997
	B	28.08	1648	1.057	0.997
	C	26.66	769.6	2.039	0.993
PFHxS	A1	101.3	8291	1.673	0.993
	A2	23.55	3366	2.081	0.995
	B	38.62	1464	2.726	0.980
GenX	A1	51.49	1963	5.488	0.985
	A2	58.33	953.1	1.285	0.989
	B	72.41	414.0	1.184	0.987
	C	56.14	1991	3.469	0.995
**PFAS**	**Biochar (PW)**	**Parameters of Freundlich model**			
		K_F_	*n*	R^2^	
PFHxS	C	5.976	1.098	0.984	

**Table 2:** Comparison between adsorption capacities (q_e_) of selected PFASs of present study and literature values.

Compounds	This Work q_e_ (mg g^−1^)			Activated Carbon (Literature) q_e_ (mg g^−1^)	Reference
	PW-B	PW-C	AC		
PFBA	30	24	33	3.88	[19]
				14.98	[20]
PFBS	82	74	76	6.99	[19]
				8.68–36.54	[21]
				39.01	[20]
PFHxA	24	26	23	18.8	[22]
				2.47–22.68	[21]
PFHxS	37	32	37	21.14–55.10	[21]
GenX	68	51	38	80	[9]

**Table 3:** Adsorption parameters of Hill (Q_max_, K_D_, n_H_, and R^2^) and Freundlich (K_F_, n, and R^2^) models for PFAS mixture tests.

PFAS	Biochar (PW)	Parameters of Hill Model			
		Q_max_ (mg g^−1^)	K_D_ (mg L^−1^)	n_H_	R^2^
**PFBA**	**B**	0.802	68.46	3.89	0.984
	**C**	0.545	92.11	4.76	0.994
**PFHxA**	**B**	1.66	26.89	2.29	0.993
	**C**	2.03	48.48	1.19	0.995
**PFAS**	**Biochar (PW)**	**Parameters of Freundlich model**			
		**K_F_**	**n**	**R^2^**	
**PFBS**	**B**	0.666	0.178	0.984	
	**C**	0.337	0.366	0.991	
**PFHxS**	**B**	0.867	0.131	0.997	
	**C**	0.657	0.192	0.995	

## Data Availability

Data will be made available on request.

## Supplementary Materials

The following supporting information can be downloaded at: https://www.mdpi.com/article/10.3390/molecules31071100/s1, Table S1. Kinetic parameters of PFBA, GenX and PFHxS (k, adsorption kinetic constant; qe, equilibrium adsorbed PFAS concentration; R2, regression coefficient); Table S2. Adsorption parameters of Hill (q max, K_d_, n_H_, and R^2^) and Freundlich (K_F_, n, and R^2^) models for target PFASs adsorbed on AC; Table S3. Chemical structures and main properties of investigated PFASs; Table S4. Characterization of the study biochars; Figure S1. Adsorption isotherms of PFBA, PFBS, PFHxA, PFHxS and GenX on AC.
