# Supplementary material for: Pinewood Biochar as an Affordable Adsorbent for Short- and Medium-Chain PFAS Removal from Contaminated Water
Source: Molecules. 2026 Mar 27;31(7):1100. doi: 10.3390/molecules31071100 (PMC13074965; doi:10.3390/molecules31071100)
Supplement: Supplementary file 1 [file molecules-31-01100-s001.zip › molecules-4185364-supplementary.pdf]

Table S1. Kinetic parameters of PFBA, GenX and PFHxS. (k, adsorption kinetic constant; q_e, equilibrium sorbed PFAS concentration; and R^2, regression coefficient).

**PFAS**	**q_e (mg g^{-1})**	**K (h^{-1})**	**R^2**
PFBA	5.55	0.137	0.980
GenX	12.2	0.313	0.989
PFHxS	9.10	0.337	0.975

Table S2. Adsorption parameters of Hill (q max, K_d, n_H, R^2) and Freundlich (K_F, n, R^2) models for target PFAS sorbed on AC.

**PFAS**	**Parameters of Hill model**			
	**Q_e**	**K_d**	**n_H**	**R^2**
**PFBA**	31.68	2367	10.44	0.981
**PFBS**	83.13	572.5	1.326	0.998
**PFHxA**	22.46	949.8	4.263	0.995
**PFHxS**	55.96	2987	1.196	0.972
	**Parameters of Freundlich model**			
	**k_F**	**n**	**R^2**	
**GenX**	3.798	0.265	0.985	

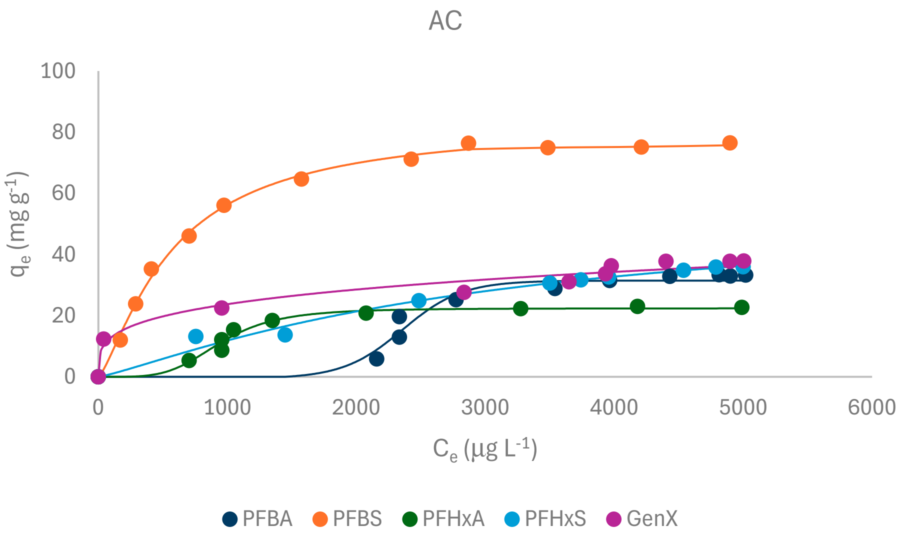

Figure S1. Adsorption isotherms of PFBA, PFBS, PFHxA, PFHxS and GenX on AC.

Table S3 Chemical structures and main properties of investigated PFAS

Compound name	Acronym	Formula	MW (g mol_1)	Functional group	Carbon chain length
Perfluorobutanoic acid	PFBA	C_4F_9COOH	214.039[a]	Carboxylic acid	C4
Perfluorobutane sulfonic acid	PFBS	$C_4F_9SO_3H$	300.095[a]	Sulfonic acid	C4
Perfluorohexanoic acid	PFHxA	$C_6F_{13}COOH$	314.054[a]	Carboxylic acid	C6
Perfluorohexane sulfonic acid	PFHxS	$C_6F_{13}SO_3H$	400.11[a]	Sulfonic acid	C6
2,3,3,3-tetrafluoro-2-(1,1,2,2,3,3,3-heptafluoropropoxy)propanoic acid	GenX	HFPO-DA	330.05 [a]	Ether carboxylic acid	branched

[a]PubChem® https://pubchem.ncbi.nlm.nih.gov/

Table S4 Characterization of the study biochars [7]

Biochar	Pyrolysis temperature	Surface area ($m^2\ g^{-1}$)	Total pore volume ($cm^3\ g^{-1}$)	Hydrodynamic size (<2R_H>)	ζ-potential (mV)	Main structural features
**PW-A1/A2**	850 °C	343 ± 2	0.383	160 ± 10 nm (40%); 680 ± 30 nm (60%)	−38 ± 3	Micro-mesoporous biochar with oxygenated functional groups
**PW-B**	1000 °C	932 ± 5	0.915	1020 ± 95 nm	−4.2 ± 0.2	Highly aromatic carbon structure with increased hydrophobicity
**PW-C**	Functionalized PW-A2	/	/	227 ± 42 nm (25%); 1010 ± 234 nm (75%)	+30 ± 1	CTAB-functionalized biochar with positively charged quaternary ammonium groups
